# Supplementary material for: (Z)-Endoxifen and Early Recurrence of Breast Cancer: An Explorative Analysis in a Prospective Brazilian Study
Source: J Pers Med. 2022 Mar 22;12(4):511. doi: 10.3390/jpm12040511 (PMC9030524; doi:10.3390/jpm12040511)
Supplement: Supplementary file 1 [file jpm-12-00511-s001.zip › jpm-1632095-supplementary.pdf]

Journal of Personalized Medicine

MDPI

Supplementary Table S1. Univariate Cox regression analyses for an association of factors with event-free survival.

Variable(s)	Value	Total (*n*)	Events [*n* (%)]	HR	95% CI	*p*
Age (years)	≥ 69	28	7 (25)	1 (Reference)		
	50–69	59	7 (11.9)	0.61	0.24–1.57	0.31
	≤49	61	11 (18)	0.38	0.13–1.08	0.07
Stage	I	50	3 (6.0)	1 (Reference)		
	II	74	12 (16.2)	2.88	0.81-10.24	0.10
	III	24	10 (41.7)	10.38	2.82–38.14	<0.001
Molecular subtype	Luminal A	48	5 (10.4)	1 (Reference)		
	Luminal B	82	16 (19.5)	2.11	0.77–5.76	0.14
	HER2	18	4 (22.2)	2.14	0.57–7.98	0.26
Ki67 (%)	≤14	49	5 (10.2)	1 (Reference)		
	>14	99	20 (20.2)	2.21	0.83-5.89	0.11
CYP2D6 phenotype class	EM/UM	97	19 (19.6)	1 (Reference)		
	IM/PM	52	6 (11.5)	0.53	0.21–1.33	0.18
Chemotherapy	No	55	8 (14.5)	1 (Reference)		
	Yes	94	17 (18.1)	1.25	0.54–2.90	0.60
BMI (kg/m^2)	≤30	61	10 (16.4)	1 (Reference)		
	>30	26	5 (19.2)	1.32	0.45–3.87	0.61
Adherence	Low	16	1 (6.3)	1 (Reference)		
	High/ Intermedi-ary	116	19 (16.4)	2.91	0.39–22.07	0.291

Supplementary Table S2. CYP2D6 alleles and their interpretation, activity scores, and CYP2D6 phenotypes.

***CYP2D6* genotypes**		**CYP2D6 phenotypes**	
Alleles (haplotypes)	Enzymatic activity of alleles	CYP2D6 activity score	CYP2D6 phenotype
*1XN	UM	UM/EM (3.0)	UM
*1, *2	EM	EM/EM (2.0)	EM
		EM/IM (1.5)	
*9, *10, *17, *29, *41	IM	EM/PM (1.0)	IM
		IM/IM (0.75)	
		PM/IM (0.5)	
*3, *4, *5, *6, *7, *8	PM	PM/PM (0.0)	PM

Abbreviations: *1XN, gene duplication; UM, ultrarapid metabolizer; EM, extensive metabolizer; IM, intermediate metabolizer; PM, poor metabolizer.

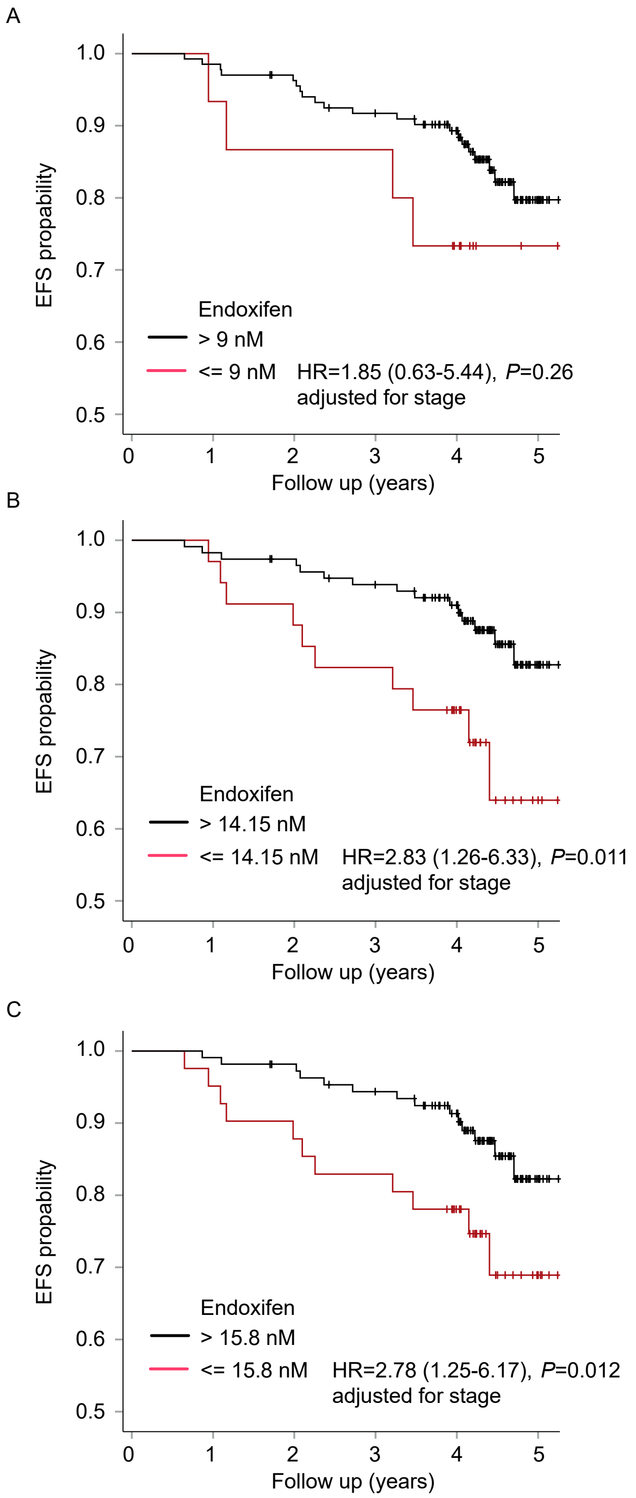

Supplementary Figure S1. Kaplan-Meier curves of (Z)-endoxifen stratified by three thresholds reported from literature. (**A**) 9 nM [10]; (**B**) 14.15 nM [9]; (**C**) 15.8 nM [11]. Hazard ratios and 95% confidence intervals (given in brackets) for below versus above threshold were calculated by multivariate Cox proportional hazard regression, adjusted for stage.
